# Co-Pyrolysis of Bamboo and Rice Straw Biomass with Polyethylene Plastic: Characterization, Kinetic Evaluation, and Synergistic Interaction Analysis

**DOI:** 10.3390/polym17152063

**Published:** 2025-07-29

**Authors:** Munir Hussain, Vikul Vasudev, Shri Ram, Sohail Yasin, Nouraiz Mushtaq, Menahil Saleem, Hafiz Tanveer Ashraf, Yanjun Duan, Muhammad Ali, Yu Bin

**Affiliations:** 1 College of Textile Science and Engineering, Zhejiang Sci-Tech University, Hangzhou 310018, China; munir88@zju.edu.cn (M.H.);; 2 National-Provincial Joint Engineering Research Center of Biomaterials for Machinery Package, Nanjing Forestry University, Nanjing 210037, China; 15951945847@139.com; 3 Department of Engineering Mechanics, Zhejiang University, Hangzhou 310027, China; 12024087@zju.edu.cn; 4 Hydrogen Energy Institute, College of Energy Engineering, Zhejiang University, Hangzhou 310027, China; sohailyasin@zju.edu.cn; 5 School of Material Science and Engineering, Zhejiang Sci-Tech University, Hangzhou 310018, China; mushtaqnouraiz@zstu.edu.cn

**Keywords:** co-pyrolysis, biomass, polyethylene, kinetics, catalysts

## Abstract

This study investigates the co-pyrolysis behavior of two lignocellulosic biomass blends, bamboo (B), and rice straw (R) with a plastic polyethylene (P). A total of 15 samples, including binary and ternary blends, were analyzed. Firstly, X-ray diffraction (XRD) analysis was performed to reveal high crystallinity in the B25R75 blend (I/I_c_ = 13.39). Whereas, the polyethylene samples showed persistent ZrP_2_O_7_ and lazurite phases (I/I_c_ up to 3.12) attributed to additives introduced during the manufacturing of the commercial plastic feedstock. In addition, scanning electron microscopy with energy-dispersive X-ray (SEM-EDX) spectroscopy was performed to characterize the surface morphology and elemental composition of the feedstock. Moreover, thermogravimetric analysis (TGA) was employed at temperatures up to 700 °C at three different heating rates (5, 10, and 20 °C/min) under pyrolysis conditions. Kinetic analysis used TGA data to calculate activation energy via Friedman’s isoconversional method, and the blended samples exhibited a decrease in activation energy compared to the individual components. Furthermore, the study evaluated transient interaction effects among the components by assessing the deviation between experimental and theoretical weight loss. This revealed the presence of significant synergistic behavior in certain binary and ternary blends. The results demonstrate that co-pyrolysis of bamboo and rice straw with polyethylene enhances thermal decomposition efficiency and provides a more favorable energy recovery route.

## 1. Introduction

Biomass is increasingly recognized as a vital resource for renewable energy production due to its abundance and cost-effectiveness. It includes diverse materials such as wood, agricultural residues, municipal solid waste, and algae [1,2]. Further, it serves as a versatile energy source, capable of being directly used for heat and power generation or converted into transportation fuels and chemical feedstocks through various thermochemical technologies [3]. Utilizing biomass can help mitigate climate change, enhance energy security, and promote rural development. It is considered carbon-neutral over its lifecycle, contributing to reduced greenhouse gas emissions [4]. Biomass can be converted into various forms of energy, including electricity, transportation fuels, and process heat for industries. This versatility makes it a crucial component in transitioning to a sustainable energy system [5,6,7]. Rice straw, a by-product of rice cultivation, is often discarded or incinerated, leading to environmental concerns despite its potential value [8]. Bamboo biomass, like rice straw, is a significant source of renewable energy [9]. In addition, different catalysts and their varying concentrations also play a major role in improving thermal degradation characteristics [10]. The pyrolysis of polyethylene is a promising method of energy recovery from waste polymers, which involves thermal decomposition of polymers resulting in a large number of hydrocarbon products [11]. The pyrolysis of polyethylene may yield a variety of products such as ethylene, propylene, and butylene which are valuable in petrochemical industries [12]. Meanwhile, the flame propagation behavior and temperature characteristics of polyethylene dust were explored by Gan et al. (2018), and it was observed that the pyrolysis starts at temperatures around 226–242 °C, while a rapid weight loss was witnessed around 400–428 °C [13].

Co-pyrolysis is a thermochemical process where two or more materials are decomposed together to produce bio-oil, syngas, and biochar [14]. Wang et al. (2022) explored thermal degradation behavior of pinewood and high-density polyethylene (HDPE) co-pyrolysis and revealed that the presence of HDPE enhanced the thermal degradation of different biomass components [15]. Also, an extra peak denoting the decomposition of HDPE was seen in the DTG curves as opposed to pinewood decomposition. The co-pyrolysis of polyethylene, cornstalk, and anthracite coal was studied by Gou et al. (2019) by employing TGA-FTIR and three different stages were observed corresponding to cornstalk, polyethylene, and anthracite coal thermal degradation [16]. Timilsina et al. (2024) explored the artificial intelligence-based optimization strategies for pyrolysis and co-pyrolysis of biomass and plastic and observed that machine learning models can predict bio-oil yield and composition with high accuracy (R^2^ > 0.97), while the uncertainty analysis revealed that the maximum probability of bio-oil yield has to be in the range of 30–50% [17]. Wang and Li (2008) also explored thermal degradation during the pyrolysis of PLA and biomass mixtures and revealed that PLA decomposes mostly in the 300–372 °C range, while major biomass devolatilization takes place in the 183–462 °C temperature range [18].

Further, TGA data is essential for evaluating the kinetic parameters of biomass pyrolysis. This includes determining activation energies (*E_α_*, kJ mol^−1^), reaction mechanisms (*f* (*α*)), and frequency factors (*A*, min^−1^), which are critical for optimizing the pyrolysis process [19]. The co-pyrolysis of polylactic acid (PLA) and sugarcane bagasse (SCB) was studied to explore the kinetic parameters and a linear relationship between *E* and ln*A* was observed for PLA and SCB blends indicating increased *A* values with an increase in *E*, which suggests that pyrolysis degradation is more difficult in later conversion (*α*) stages [20]. Also, the effect of an acid mine drainage (AMD) catalyst on the kinetic parameters during the co-pyrolysis of spent coffee (SC) grounds and high-density polyethylene (HDPE) was discussed by Bhushan et al. (2024), and it was found that the activation energy is reduced by 16.95% in the catalytic as opposed to the non-catalytic cases and that pre-exponential factor values also decreased [21]. Ram et al. (2024) performed kinetic analysis using Friedman’s isoconversion method during thermogravimetric combustion and revealed that algae biochar requires greater activation energies as opposed to lignocellulosic biochar [22]. Also, kinetic analysis and experimental data prediction using an artificial neural network (ANN) was described for the combustion and pyrolysis of dairy waste, and it was observed that the ANN is capable of predicting non-linear relationships among temperature, heating rate, and weight loss without any mathematical prescription [23]. Wang et al. (2024) combined the multicomponent Gaussian kinetic modeling and ANN analysis for the pyrolysis of macadamia nut peel and revealed that ANN modeling provided a robust and reliable framework for predicting thermal degradation characteristics, complementing the insights of thermo-kinetic analysis [24].

Subsequently, co-pyrolysis of different biomass types, such as terrestrial and aquatic biomass, can exhibit synergistic effects that enhance overall process efficiency. These effects can be analyzed using specific methods like overlap ratio (OR) and the difference between experimental and theoretical weight (Δ*W*) values [25]. Liu et al. (2021) put in effort to analyze the synergistic effect during the co-pyrolysis of pinewood and polycarbonate and observed a significant synergistic effect leading to enhanced energy recovery and improved waste valorization [26]. Thermal degradation characteristics during co-pyrolysis were studied by Yuan et al. (2024), and it was revealed that corn straw (CS) mixed with bituminous coal (BC) in a 30:70 blending ratio exhibited the highest positive synergistic effect [27]. Also, Chen et al. (2024) explored the synergistic effect between lignin and plastic mixtures during catalytic pyrolysis in the presence of the HZSM-5 catalyst and found that the interaction between lignin and low-density polyethylene resulted in enhanced HC formation in the form of increased hydrogen transfer and Diels–Alder reactions [28]. Further, the effect of temperature and blending ratio during co-pyrolysis of biomass and coal revealed that the increasing biomass ratios lead to increased bio-oil yield concerning the synergistic interactions and physicochemical characteristics of coal and biomass [29].

While previous studies have extensively investigated biomass co-pyrolysis, significant gaps remain in understanding the catalytic role of polyethylene and its synergistic interactions with bamboo and rice straw. Existing kinetic studies often overlook the combined effects of feedstock blending and catalytic enhancement, particularly for these biomass types. To address these gaps, this study systematically examines the thermal degradation behavior of bamboo–rice straw–polyethylene blends via thermogravimetric analysis (TGA). In addition, the activation energy of the process was calculated using Friedman’s isoconversional method. Moreover, synergistic effects were quantified through the calculation of a weight loss-based parameter. By integrating SEM-EDX, XRD, and morphological analyses, we further elucidated the role of inorganic constituents in catalytic interactions. This study builds on previous research on biomass–polyethylene co-pyrolysis by incorporating kinetic modeling alongside mineralogical and surface analyses to quantify synergistic interactions and understand the role of specific inorganic components. In contrast to earlier investigations, we examine the influence of potassium-rich biomass and inert mineral phases in polyethylene on thermal degradation pathways within binary and ternary feedstock combinations.

## 2. Materials and Methods

### 2.1. Samples

In the present study, two different lignocellulosic biomass feedstocks, namely bamboo and rice straw (RS), and one plastic sample, polyethylene, were used. The biomass and plastic samples were collected from a local marketplace in Hangzhou, China. After collection, the biomass samples were washed with distilled water to wash off surface dust and contaminants and then dried in an oven at 105 °C for 24 h. The dry biomass samples were then ground and sieved to maintain a particle size less than 450 μm. The polyethylene samples were already received in powdered form with particle sizes less than 50 μm. Afterwards, three different blending ratios of 25:75, 50:50, and 75:25 were used to prepare binary mixtures between BM, PE, and RS, thereby generating a total of 9 binary blend samples. Meanwhile, three ternary blends with ratios of 25:25:50, 25:50:25, and 50:25:25 were prepared for the three samples, respectively. Hence, a total of 12 blended and 3 neat samples were analyzed in this study. All the samples were abbreviated by a common rule, i.e., the first letter of the sample name followed by its corresponding ratio in the blend. For instance, neat samples of bamboo, rice straw, and polyethylene were named B100, R100, and P100, respectively. A 25:75 binary blend of bamboo and polyethylene is abbreviated as B25P75. Similarly, a ternary blend of 25:50:25 of bamboo, rice straw, and polyethylene, respectively, is abbreviated B25R50P25.

### 2.2. Experimental Procedures

#### 2.2.1. Characterization

The raw biomass and polyethylene samples were characterized using scanning electron microscopy with energy dispersive X-ray (JSM-5610LV SEM, JEOL Company, Tokyo, Japan) and X-ray diffraction (ARL X’TRA X-ray powder diffractometer, Thermo Electron Corp., Waltham, MA, USA) analysis in the 2θ-angle range of 10–80°. These results were further analyzed to detect mineral phases by using QualX2.0 software equipped with the crystallography open database (COD).

#### 2.2.2. Pyrolysis Experiments

Non-isothermal pyrolysis experiments were performed in micro-scaled thermogravimetric analysis (TGA) equipment (TA Instruments, New Castle, DE, USA). Approximately 5 mg of sample was taken to perform the pyrolysis experiments. The temperature was raised from room temperature to 700 °C at three different heating rates of 5, 10, and 20 °C/min, and maintained at 700 °C for 5 min. During these experiments, high-purity nitrogen was purged at a flow rate of 100 mL/min to create an inert atmosphere.

### 2.3. Kinetic Modelling

Weight loss data is normalized to obtain the conversion (*α*) parameter

(1) $$\alpha =\frac{w_{i}-w_{t}}{w_{i}-w_{f}}$$

where *w_i_*, *w_t_*, and *w_f_* represent the initial, instantaneous, and final weight values, respectively. Note that, *w_i_* and *w_f_* for the kinetic study were taken in the major devolatilization temperature range, i.e., 150–600 °C. In solid-state kinetics, *α* is dependent on temperature (*T*) and [30,31,32,33]

(2) $$\frac{d\alpha }{dT}=\frac{A}{\beta }\exp\left(\frac{-E}{RT}\right)f\left(\alpha \right)$$

Here, *R* is a universal gas constant and *β* is the heating rate. Further rearrangement of Equation (2) provides the following correlation [34,35]:

(3) $$\ln\left(\beta \frac{d\alpha }{dt}\right)=\ln\left[Af(\alpha )\right]-\frac{E}{RT}$$

In this equation, *E* is determined using the slope between *ln*[*β*(*dα*/*dt*)] and 1/*T* at various *α* and at least three *β* values [36]. This approach assumes that the governing mechanism is an order-based reaction model [37,38]

(4) $$f\left(\alpha \right)={\left(1-\alpha \right)}^{n}$$

The advantage of the machine learning-based kinetic analysis model is that it gives the kinetic triplet values for each heating rate with higher accuracy.

### 2.4. Interaction Analysis

Interaction between the samples within the blends during pyrolysis was evaluated based on the mass loss data. The deviation (Δ*W*) between experimentally observed (*W*_exp_) and calculated (*W*_cal_) weight was used for analyzing this synergistic interaction [39,40]

(5) $$W_{cal}=x_{B}W_{B}+x_{P}W_{P}+x_{R}W_{R}$$

(6) $$\Delta W=W_{cal}-W_{\exp}$$

here, *x*_B_, *x*_P_, and *x*_R_ represent the blending ratios of bamboo, plastic, and rice straw, respectively, while *W*_B_, *W*_P_, and *W*_R_ signify the weight values during the pyrolysis of bamboo, plastic, and rice straw, respectively. A positive value of Δ*W* signifies that the experimentally measured weight loss exceeds the theoretically predicted value based on the weighted average (Equation (4)). This deviation suggests a synergistic interaction between the blended components, resulting in enhanced volatile matter release during thermal decomposition.

## 3. Results and Discussion

### 3.1. Mineral Analysis Using XRD

**Figure 1:**
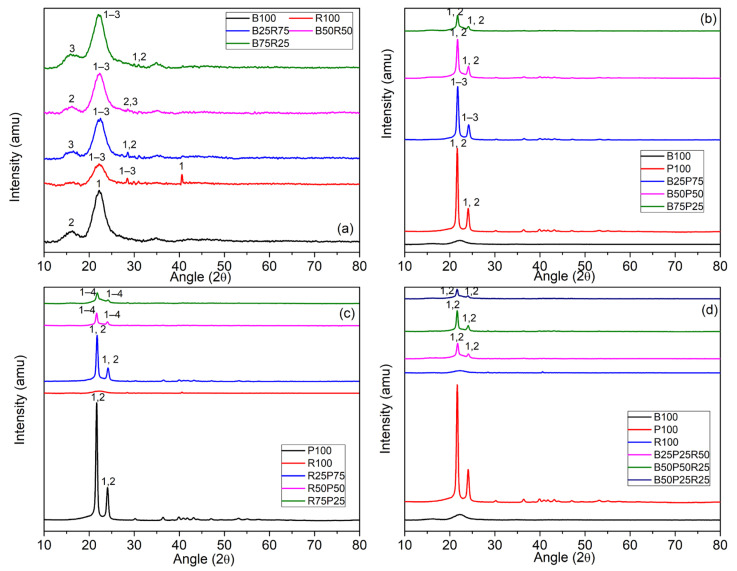
XRD patterns for (**a**) bamboo, rice straw, and their binary blends; (**b**) bamboo, polyethylene, and their binary blends; (**c**) polyethylene, rice straw, and their binary blends; and (**d**) bamboo, polyethylene, rice straw, and their ternary blends. The numbers (1–4) indicate the occurrence of different crystallographic phases, as detailed in Table 1.

X-ray diffraction (XRD) analysis (Figure 1 and Table 1) was conducted to investigate the crystalline structures and mineral compositions of the raw (un-pyrolyzed) forms of bamboo biomass (B), rice straw biomass (R), polyethylene plastic (P), and their various binary and ternary blends. Note that the labels 1, 2, 3, and 4 in Figure 1a–d denote the names of phases present on particular intensity peaks, as listed in Table 1. In the raw bamboo sample (B100), two crystalline phases were identified: SiO_2_ (quartz), with a major peak at 22.15° (2θ) and the intensity ratio (I/I_c_) of 1.21, associated with the (2 −1 0) plane in a triclinic crystal system; and C_8_H_7_MnO_3_, a monoclinic organometallic compound with a peak at 16.17° (I/I_c_ = 0.99). These reflect bamboo’s native silica content and traces of metal–organic complexes absorbed from its growing environment. The raw rice straw sample (R100) showed a more complex mineral profile, with dominant phases including C_42_H_30_Na_6_O_12_ (40.55°, I/I_c_ = 2.38), NaNO_3_ (22.68°, I/I_c_ = 2.28), and SiO_2_ (22.59°, I/I_c_ = 3.33), exhibiting triclinic and trigonal systems. These patterns are consistent with rice straw’s high ash content and its tendency to accumulate alkali and silica-based minerals during growth. In the case of polyethylene plastic (P100), the XRD patterns revealed crystalline residues likely originating from additives or contaminants in the commercial plastic. Major peaks corresponded to ZrP_2_O_7_ at 21.55° (0 6 0) with I/I_c_ = 1.56, and lazurite at 24.05° (2 0 6), both featuring orthorhombic crystal systems. These phases appeared consistently across plastic-containing blends, indicating their stable crystalline nature even before thermal exposure.

Among the binary biomass blends, B25R75 exhibited the highest peak intensity (I/I_c_ = 13.39) at 19.97°, corresponding to a trigonal phase of a Ce–Mn–I-based compound [(CeI)_0_._12_(Ce_6_MnI_9_)], with a high estimated crystallographic density of 5.35 g/cm^3^. In plastic-rich blends, such as B25P75, B50P50, B75P25, R25P75, R50P50, and R75P25, recurring peaks of ZrP_2_O_7_ and lazurite were observed, indexed to planes like (0 6 0), (0 6 3), and (2 5 4), with I/I_c_ values reaching up to 3.12. These orthorhombic structures reflect the influence of plastic additives on the mineral fingerprint of the raw mixtures.

In ternary blends such as B25P25R50, plastic-derived crystalline phases remained dominant, whereas the B25P50R25 blend showed additional peaks for Li (AlSi_4_O_10_), a monoclinic aluminosilicate phase.

Other combinations like B50R50 and B75R25 displayed a variety of mineral and organo-metallic compounds, including Mo_8_O_44_P_8_, C_12_H_18_N_4_O_3_, and C_15_H_3_CrF_18_O_6_, mostly in monoclinic form and with moderate intensities (I/I_c_ ≤ 4.97), reflecting the compositional diversity of unprocessed biomass mixtures.

Crystallographic density estimations further indicated that samples with high plastic content or rare-earth phases (e.g., B25R75) had elevated densities (up to 5.91 g/cm^3^), while biomass-only samples such as B100 and R100 displayed lower densities in the range of 1.67 to 3.36 g/cm^3^.

Note that the emergence of “new” crystalline phases in the XRD patterns of blends may arise from (1) the superposition of weak trace minerals in bamboo and rice straw whose overlapping reflections become detectable at a specific ratio and/or (2) preferred orientation of crystallites induced by vigorous dry mixing, which amplifies specific diffraction peaks.

### 3.2. SEM-EDX Characterization

**Figure 2:**
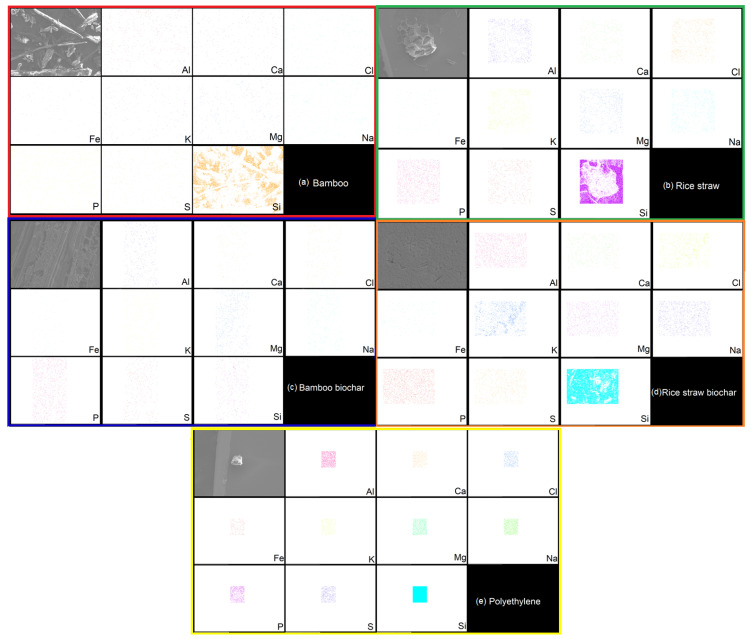
SEM-EDX analysis of individual samples: (**a**) bamboo (B100), (**b**) rice straw (R100), (**c**) bamboo biochar, (**d**) rice straw biochar, and (**e**) polyethylene (P100).

Figure 2 displays the SEM-EDX micrographs with a scale bar of 200 μm, confirming the presence of several major elements in the B100, P100, and R100 samples. Table 2 summarizes the atomic and weight percentages of these elements. B100 (Figure 2a) exhibited the presence of Si (26.93 wt%), along with significant contributions from C (48.36 wt%) and O (20.77 wt%). P100 showed a dominant presence of Si (87.61 wt%), with minor traces of C (7.26 wt%) and O (1.79 wt%). R100 (Figure 2c) contained Si (30.24 wt%), C (43.22 wt%), and O (19.02 wt%), along with trace amounts of Cl (1.03 wt%), and K (1.61 wt%). Notably, Si was primarily concentrated in the outer regions of the micrograph. Additionally, SEM-EDX analysis was performed on bamboo biochar and rice straw biochar. Bamboo biochar was predominantly composed of C (88.60 wt%) and O (5.10 wt%), with minor traces of Zr (3.55 wt%), Au (1.36 wt%), S (0.43 wt%), Mg (0.28 wt%), and Si (0.25 wt%). Rice straw biochar contained C (35.31 wt%), O (5.86 wt%), and Si (48.55 wt%), along with trace elements such as S (0.35 wt%), Cl (1.48 wt%), and K (3.34 wt%).

Notably, the biochar samples demonstrated significant compositional differences where bamboo biochar was highly carbon-rich (88.60 wt% C) with negligible Si content, whereas rice straw biochar retained a substantial Si fraction (48.55 wt%) alongside low carbon and oxygen. These findings suggest that feedstock type strongly influences inorganic residue distribution, which may impact subsequent thermochemical processing and ash-related challenges. The presence of Si-rich phases in certain samples could affect catalytic behavior as well as slagging tendencies during high-temperature applications. The accumulation of stable silica-based minerals in biochars significantly increases the risk of slagging and fouling in thermochemical processing systems [41]. The rice straw biochar analyzed in this work, which retained a substantial Si fraction as demonstrated by the SEM-EDX and XRD analyses, exemplifies this issue.

### 3.3. Morphological Analysis of Biomass and Its Biochar

**Figure 3:**
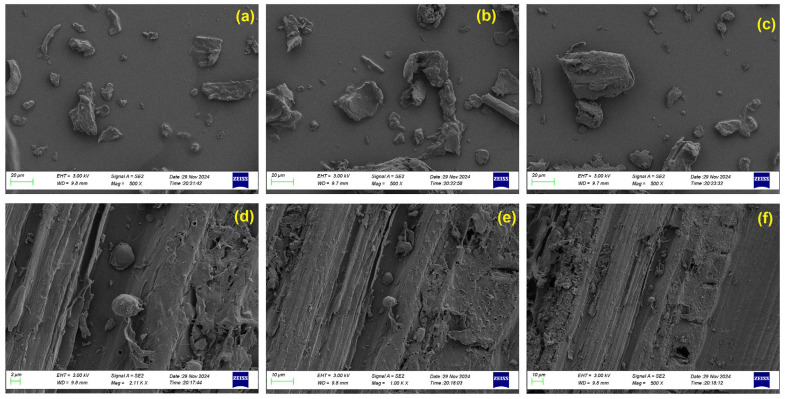
SEM images of (**a**–**c**) bamboo (B100) and (**d**–**f**) bamboo biochar.

The scanning electron microscopy (SEM) images in Figure 3a–c depict the surface morphology of raw bamboo in powdered form (particle size < 450 µm), whereas the images in Figure 3d–f illustrate the corresponding morphology of bamboo biochar obtained after pyrolysis. The raw bamboo particles exhibit relatively smooth, dense surfaces, characteristic of intact lignocellulosic biomass. Minimal surface porosity is observed, indicating the preservation of the cell wall architecture. In contrast, the bamboo biochar particles demonstrate significant morphological changes induced by thermal decomposition. The surfaces of biochar appear rough, fragmented, and porous, reflecting the volatilization of the organic constituents and the structural reorganization occurring during pyrolysis. Notably, the biochar exhibits a network of pores, which likely emerged due to the diffusion of volatile gases during the breakdown of hemicellulose and cellulose.

**Figure 4:**
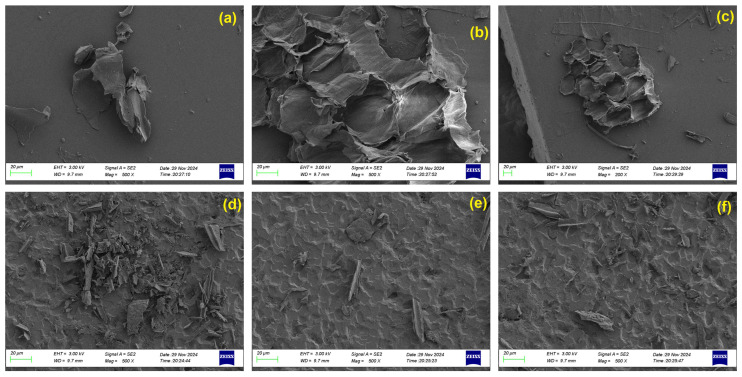
SEM images of (**a**–**c**) rice straw (R100) and (**d**–**f**) rice straw biochar.

The SEM images of raw rice straw particles in Figure 4a–c show flaky, irregularly shaped structures with a layered or sheet-like morphology. The surface texture is relatively smooth with occasional fibrous elements and minor cracking, likely from sample preparation. There is little evidence of internal porosity, and the surfaces appear mostly compact and intact. After pyrolysis, the rice straw biochar (d–f) demonstrates a dramatically different morphology. The particles have undergone significant fragmentation and surface roughening. Multiple pores, voids, and flakes are clearly visible across the surface, along with a more heterogeneous texture. The overall appearance is more brittle and porous compared to the raw rice straw, reflecting the volatile release and thermal decomposition of organic constituents. These microstructural changes enhance the surface area and may contribute to improved adsorptive or catalytic potential [42].

### 3.4. Thermogravimetric Analysis

**Figure 5:**
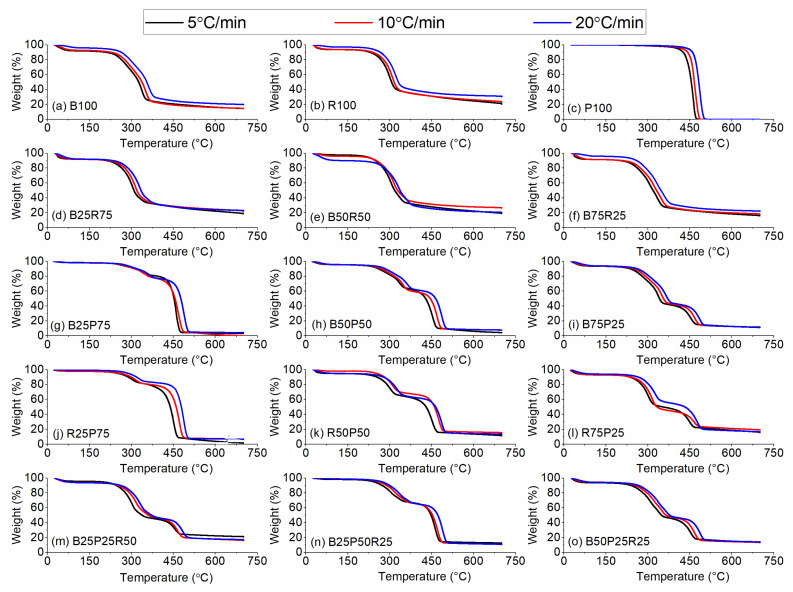
Thermogravimetric (TG) thermal degradation curves.

**Figure 6:**
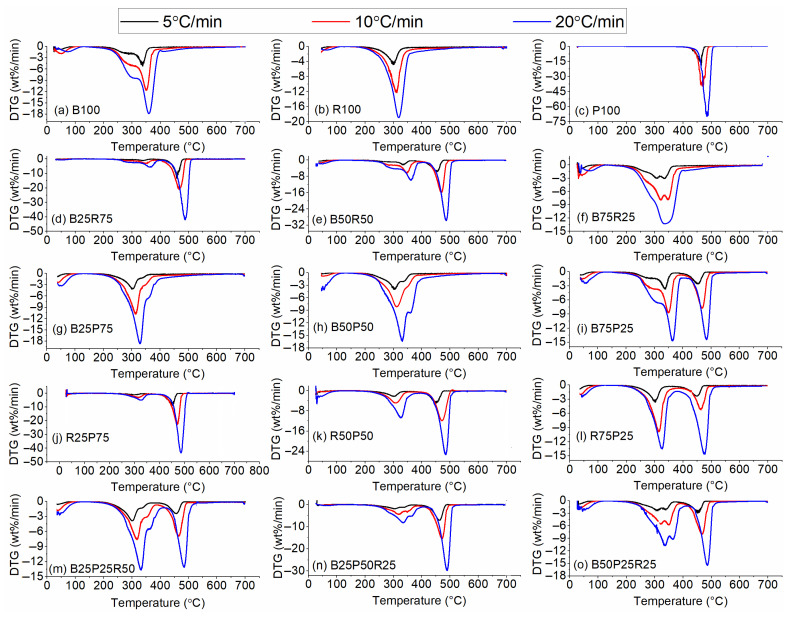
Differential thermogravimetric (DTG) curves.

Figure 5a–o and Figure 6a–o represent the TG and DTG curves during pyrolysis and co-pyrolysis of raw and blended (i.e., binary and ternary) samples, respectively. The early weight losses with increased temperatures (i.e., up to 150 °C) denote the elimination of moisture and light volatiles, while the mass loss peaks observed in the range of 150–600 °C represent the devolatilization of volatiles, which is often termed a major devolatilization region. For instance, the smaller peaks on the left side, nearly up to 150 °C in Figure 6a, indicate the moisture removal at the different heating rates employed. In addition, the major peak represents the thermal decomposition of hemicellulose and cellulose. In the later stage of thermal decomposition, lignin decomposition leading to char formation took place. Subsequently, the thermal decomposition of R100 and P100 occurred in similar fashion as witnessed in Figure 6b,c. Additionally, increased heating rates increased the maximum thermal degradation rates which shifted the major devolatilization peaks towards greater temperatures. Comparatively, P100 exhibited the highest thermal degradation rates as opposed to B100 and P100. Also, the increased heating rates resulted in increased solid residue values for B100 and R100, while P100 decomposed completely leaving behind no solid residues, at all heating rates tested (refer to Figure 5a–c).

Subsequently, co-pyrolysis of binary and ternary blends of bamboo, polyethylene, and rice straw showed obvious changes in thermal degradation characteristics. For example, when bamboo was mixed with rice straw in three different ratios (i.e., 25%, 50%, and 75%), an extra shoulder on the right side of the major devolatilization peak appeared. This peak becomes more prominent for B50R50 and B75R25. Further, introducing polyethylene to the bamboo and rice straw showed greater degradation rates owing to polyethylene having the highest devolatilization rates during the thermal decomposition of raw samples. In addition, PE and biomass blends exhibited two separate degradation peaks in the major devolatilization regime, while decreasing PE concentrations led to decreased thermal degradation rates. Furthermore, adding polyethylene to bamboo and rice straw decreased the solid residue values as opposed to the pyrolysis-derived solid residues of rice straw and bamboo. Nevertheless, increasing heating rates increased the solid residues for all the sample blends, with few notable exceptions, as seen in Figure 6. In addition, co-pyrolysis of bamboo and rice straw did not exhibit the obvious effects on the final solid residue at different blending ratios. In addition, the ternary blends display a clear shift in the onset temperature of major devolatilization compared to both binary and single-component samples, indicating that the presence of all three feedstocks alters the thermal stability of the mixture. Notably, the B25P50R25 blend (Figure 6n) exhibits not only the highest peak degradation rate but also a broadened devolatilization zone, reflecting simultaneous breakdown of the cellulose, hemicellulose, and PE chains. Moreover, the final char yields of the ternary mixtures fall between those of the pure biomass and pure plastic samples, with blends containing higher PE fractions producing proportionally lower residues, confirming the moderating effect of polyethylene on overall char formation.

### 3.5. Kinetic Analysis

**Figure 7:**
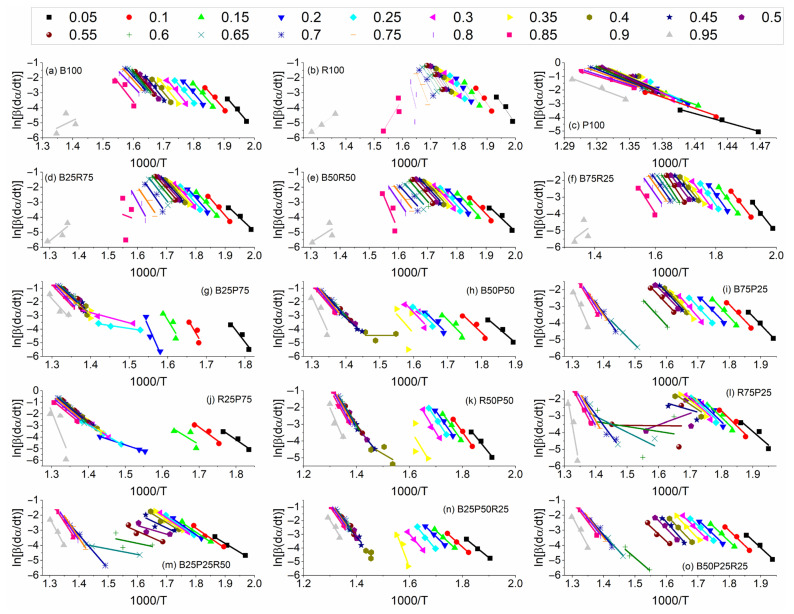
Linear isoconversional plots obtained using Friedman equation.

Figure 7 presents the linear isoconversional plots generated using the Friedman method for the thermal degradation of the individual and blended feedstocks comprising bamboo (B), rice straw (R), and polyethylene (P). The plots illustrate the dependence of the logarithmic rate expression, ln[*β*(*dα*/*dt*)], on the reciprocal temperature (1000/*T*), evaluated across a series of conversion degrees (*α* = 0.05–0.95). Table 3 lists the *E*_α_ values calculated from the slopes of the linear fits. Notably, samples such as P100, R100, and most blends with significant PE or rice straw content exhibit pronounced variation and irregularity in their Friedman plots and activation energies across the conversion range, indicating complex, multi-step reaction mechanisms. Polyethylene pyrolysis proceeds through overlapping radical-driven stages such as initial high-energy random chain scission, β-scission and hydrogen transfer propagation, secondary fragmentation of low-molecular-weight oligomers, and diffusion-limited radical recombination [43]. These stages may result in a conversion-dependent activation energy profile that rises during backbone cleavage and falls as shorter fragments decompose. R100 similarly reveals large *E*_α_ variation, reflecting the sequential degradation of hemicellulose, cellulose, and then lignin/mineral phases in rice straw. Blends rich in PE or rice straw (e.g., B25P75 or R50P50) display comparable multi-stage behavior, as indicated by the divergence and crossing of their conversion plots. In contrast, B100 and bamboo-rich blends (e.g., B75P25 or B50P50) display relatively parallel and tightly grouped plots with minor *E*_α_ fluctuations, consistent with simpler, more uniform pyrolysis behavior. Overall, these results indicate that the presence of bamboo in blends tends to reduce kinetic complexity.

**Figure 8:**
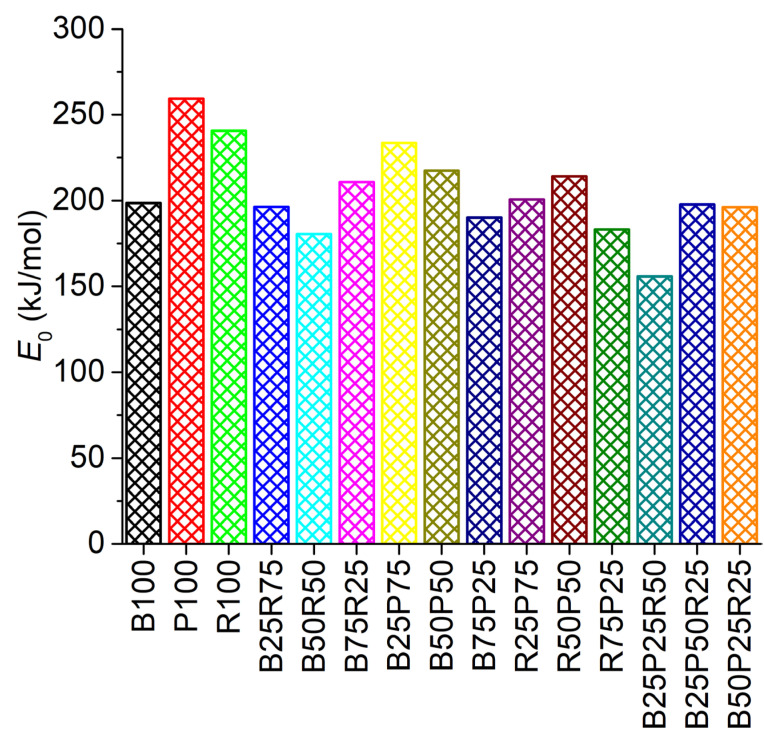
Average activation energy (*E*_0_) variations for raw, binary, and ternary sample blends.

As shown in Figure 8, the kinetic analysis of co-pyrolysis blends revealed significant variations in average activation energy (*E*_0_) values, reflecting the complex interplay between feedstock composition and thermal degradation behavior. Pure polyethylene (P100) exhibited the highest *E*_0_ (259.28 kJ/mol), consistent with its stable hydrocarbon structure requiring substantial energy for chain scission. In contrast, the lower *E*_0_ values for bamboo (B100, 198.57 kJ/mol) and rice straw (R100, 240.59 kJ/mol) correlate with their lignocellulosic structures, where hemicellulose and cellulose decompose at relatively lower energies [44]. For binary blends, the bamboo–rice straw samples demonstrated a clear trend of increasing *E*_0_ with higher bamboo content (B25R75: 196.34 kJ/mol → B75R25: 210.78 kJ/mol). This progression can be attributed to bamboo’s higher lignin content, which decomposes over a broader temperature range and requires greater activation energy compared to rice straw. In polyethylene–bamboo blends, the *E*_0_ values (190–234 kJ/mol) were intermediate between pure polyethylene and bamboo. Notably, the presence of alkali metals in rice straw, such as potassium (see Table 2), likely promoted bond cleavage in polyethylene. Therefore, the *E*_0_ values of the polyethylene–rice straw blends were lower than both the individual pure samples. The ternary blend B25P50R25 exhibited the highest *E*_0_ (197.74 kJ/mol) among the ternary samples, indicating dominant thermal stability at a high loading (50%) of polyethylene. Conversely, the ternary blend with a higher rice straw ratio (B25P25R50, *E*_0_ = 155.89 kJ/mol) again reduced the activation energy of the blend, possibly due to the catalytic effect of potassium. The blend with more bamboo loading (B50P25R25, *E*_0_ = 196.24 kJ/mol) was close to the sample B25P50R25.

### 3.6. Synergistic Effects

**Figure 9:**
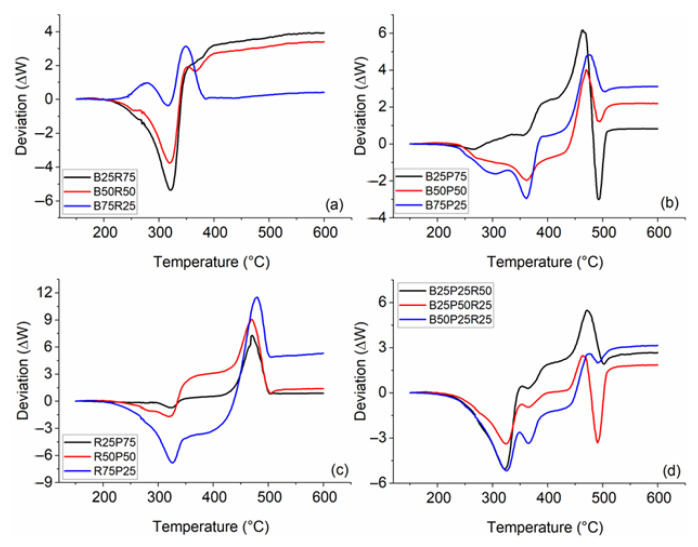
Interaction analysis for the blends of (**a**) bamboo and rice straw; (**b**) bamboo and polyethylene; (**c**) rice straw and polyethylene; and (**d**) bamboo, polyethylene, and rice straw at 20 °C/min.

The interaction analysis for the pyrolysis blends at 20 °C/min is presented in Figure 9. The observed synergistic interactions between biomass components and polyethylene in this study are consistent with findings reported in the existing literature [45]. In this analysis, negative Δ*W* values indicate negative synergistic effects, while positive Δ*W* values represent positive synergy between the sample blends. For the co-pyrolysis of bamboo and rice straw (Figure 9a), the B75R25 blend exhibited a consistent positive synergy throughout the devolatilization process, as reflected by positive Δ*W* values. In contrast, the B25R75 and B50R50 blends showed negative Δ*W* values up to approximately 350 °C, after which they transitioned to positive values in the later stages of pyrolysis. When bamboo and polyethylene were blended (Figure 9b), the B25P75 blend maintained positive Δ*W* values across most of the temperature range, only turning negative around 500 °C. Both the B50P50 and B75P25 blends started with negative Δ*W* values but eventually became positive as pyrolysis progressed. For the co-pyrolysis of rice straw and polyethylene (Figure 9c), all blends, except R75P25, exhibited positive Δ*W* values throughout the entire temperature range, indicating a consistent positive synergistic effect. The R75P25 blend, however, demonstrated negative Δ*W* values during the initial phase of pyrolysis before shifting to positive values later on. Lastly, for the ternary blends of bamboo, rice straw, and polyethylene (Figure 9d), all mixtures initially showed negative Δ*W* values up to around 350 °C, followed by a transition to positive Δ*W* values, indicating positive synergistic interactions in the later stages of pyrolysis. Mechanistically, this shift arises because PE begins main-chain scission and β-scission around 380–420 °C, generating radicals and low-molecular-weight fragments that accelerate biomass devolatilization. In PE blends, it is noteworthy that the synergy not only becomes positive but does so to a significantly greater extent than in biomass-only blends. Simultaneously, the alkali and alkaline-earth metals (AAEMs) present in rice straw, such as K and Na, can catalyze dehydration and ring-fission reactions. When radicals derived from polyethylene (PE) interact with biomass ash, these AAEM-catalyzed pathways further augment mass loss. Thus, the combined radical transfer and catalytic effects explain the negative-to-positive Δ*W* transition in ternary blends above 350 °C.

## 4. Conclusions

Co-pyrolysis of binary and ternary blends of bamboo, rice straw, and polyethylene was performed in this work. XRD analysis showed that rice straw–bamboo blends (e.g., R25B75) enhanced crystalline intensity via mineral synergy, while polyethylene-containing samples consistently exhibited stable additive-derived phases like ZrP_2_O_7_ and lazurite. The SEM-EDX results revealed that bamboo biochar was highly carbon-rich (88.60 wt% C) with a low Si content, while rice straw biochar retained a substantial Si fraction (30.24 wt%), highlighting feedstock-dependent differences in biochar composition. The DTG profiles showed that the co-pyrolysis of rice straw and polyethylene resulted in a more uniform degradation rate and broadened the temperature range, which could lead to higher yields of volatile products and reduce the formation of unwanted by-products. The co-pyrolysis of polyethylene with rice straw significantly lowered the activation energy (E_0_ = 155.89 kJ/mol) compared to pure polyethylene (259.28 kJ/mol), likely due to catalytic effects from potassium-rich rice straw promoting polymer bond cleavage. Most polyethylene-biomass blends showed positive synergy during pyrolysis, with ternary and some binary blends shifting from negative to positive interactions above 350 °C. These findings suggest that such blends can enhance thermal efficiency and volatile product yields in large-scale pyrolysis reactors, but operational factors including feedstock mixing, heat/mass transfer, and management of residual ash phases, including Si-rich species must be addressed to fully realize these benefits during continuous operation.

## Figures and Tables

**Table 1:** Parameters calculated in XRD mineral analysis of all the individual and blended samples.

Sample	Phase Number	Mineral Composites	Maximum Angle (2θ)	Intensity Ratio (I/I_c_)	Diffraction Plane (hkl)	Density (g/cm^3^)	Crystal System
B100	1	SiO_2_	22.15°	1.21	(2 −1 0)	3.36 ± 0.02	Triclinic
	2	C_8_H_7_MnO_3_	16.17°	0.99	(1 1 −1)	1.67 ± 0.03	Monoclinic
P100	1	ZrP_2_O_7_	21.55°	1.56	(0 6 0)	3.14 ± 0.02	Orthorhombic
	2	Lazurite	24.05°	0.87	(2 0 6)	2.40 ± 0.05	Orthorhombic
R100	1	C_42_H_30_Na_6_O_12_	40.55°	2.38	(1 2 6)	1.52 ± 0.06	Triclinic
	2	NaNO_3_	22.68°	2.28	(1 0 −2)	2.20 ± 0.02	Trigonal
	3	SiO_2_	22.59°	3.33	(1 0 0)	3.24 ± 0.04	Trigonal
B25R75	1	KCa(H_1.764_F_1.236_)	28.46°	3.40	(2 0 0)	1.99 ± 0.01	Orthorhombic
	2	(CeI)_0.12_(Ce_6_MnI_9_)	28.51°	13.39	(3 −2 −2)	5.35 ± 0.05	Trigonal
	3	C_0.25_I_3_N_0.25_Ne_1.412_Pb	15.81°	4.57	(0 0 2)	5.91 ± 0.01	Orthorhombic
B50R50	1	Na_0.24_K_0.76_NbO_3_	22.36°	0.19	(1 0 0)	3.35 ± 0.04	Orthorhombic
	2	Mo_8_O_44_P_8_	16.43°	4.97	(2 1 −2)	3.47 ± 0.03	Monoclinic
	3	BaCa(CO_3_)_2_	28.35°	3.40	(1 1 −1)	3.67 ± 0.02	Monoclinic
B75R25	1	C_12_H_18_N_4_O_3_	22.41°	0.65	(0 0 4)	1.31 ± 0.01	Monoclinic
	2	2(C_17_H_15_N_2_OP)H_2_O	15.21°	0.74	(1 1 0)	1.35 ± 0.03	Monoclinic
	3	C_15_H_3_CrF_18_O_6_	22.31°	0.71	(1 2 3)	2.08 ± 0.04	Monoclinic
B25P75	1	ZrP_2_O_7_	24.13°	1.56	(0 6 3)	3.14 ± 0.03	Orthorhombic
	2	Lazurite	24.15°	0.87	(2 2 1)	2.40 ± 0.07	Orthorhombic
	3	Nb_2_O_15_P_4_	21.74°	1.98	(1 −2 −2)	3.18 ± 0.03	Triclinic
B50P50	1	ZrP_2_O_7_	24.12°	1.56	(2 5 4)	3.14 ± 0.06	Orthorhombic
	2	Lazurite	24.15°	0.87	(2 2 1)	2.40 ± 0.02	Orthorhombic
B75P25	1	ZrP_2_O_7_	24.13°	1.56	(0 6 3)	3.14 ± 0.04	Orthorhombic
	2	Lazurite	21.78°	0.26	(0 3 2)	2.38 ± 0.06	Triclinic
R25P75	1	ZrP_2_O_7_	24.13°	1.56	(0 6 3)	3.14 ± 0.06	Orthorhombic
	2	Lazurite	21.78°	0.26	(0 3 2)	2.38 ± 0.05	Triclinic
R50P50	1	ZrP_2_O_7_	21.55°	1.56	(0 6 0)	3.14 ± 0.04	Orthorhombic
	2	Lazurite	24.02°	0.26	(2 2 0)	2.38 ± 0.03	Triclinic
	3	AlLiO10Si_4_	24.23°	1.31	(2 0 1)	2.38 ± 0.01	Monoclinic
	4	2(C_32_H_12_BF_24_)C_24_H_48_FeO_6_ 3(C_4_H_8_O)	21.60°	0.60	(4 3 −3)	1.54 ± 0.05	Monoclinic
R75P25	1	ZrP_2_O_7_	24.13°	1.56	(0 6 3)	3.14 ± 0.05	Orthorhombic
	2	2(C_11_H_9_NS)C_5_H_8_O_4_	24.10°	1.65	(0 2 0)	1.33 ± 0.03	Monoclinic
	3	(CH_3_)_4_NClO_4_	21.65°	1.64	(2 0 1)	1.45 ± 0.03	Orthorhombic
	4	(C_4_H_9_)_4_N 1+, C_2_HO_4_ 1−, 2CS (NH_2_)_2_	21.66°	0.76	(2 0 2)	1.14 ± 0.06	Monoclinic
B25P25R50	1	ZrP_2_O_7_	24.11°	1.56	(4 2 5)	3.14 ± 0.02	Orthorhombic
	2	Lazurite	24.02°	0.26	(2 2 0)	2.38 ± 0.01	Triclinic
B25P50R25	1	ZrP_2_O_7_	21.55°	1.56	(0 6 0)	3.14 ± 0.05	Orthorhombic
	2	Li(AlSi_4_O_10_)	24.21°	1.28	(2 0 −2)	2.40 ± 0.02	Monoclinic
B50P25R25	1	ZrP_2_O_7_	21.55°	1.56	(0 6 0)	3.14 ± 0.04	Orthorhombic
	2	Lazurite	24.01°	0.87	(1 3 3)	2.40 ± 0.06	Orthorhombic

**Table 2:** Atomic percentages and weight percentages of different elements in B100, P100, and R100 samples.

Elements	Bamboo (B100)		Rice Straw (R100)		Polyethylene (P100)		Bamboo Biochar		Rice Straw Biochar	
	Weight (%)	Atom (%)	Weight (%)	Atom (%)	Weight (%)	Atom (%)	Weight (%)	Atom (%)	Weight (%)	Atom (%)
C	48.36	63.87	43.22	60.39	7.26	15.69	88.60	94.74	35.31	56.54
O	20.77	20.60	19.02	19.95	1.79	2.90	5.10	4.09	5.86	7.05
Mg	-	-	-	-	-	-	0.28	0.15	-	-
Si	26.93	15.21	30.24	18.07	87.61	80.97	0.25	0.11	48.55	33.25
S	-	-	-	-	-	-	0.43	0.17	0.35	0.21
Cl	-	-	1.03	0.49	-	-	-	-	1.48	0.80
K	-	-	1.61	0.69	-	-	0.43	0.14	3.34	1.64
Pt	3.94	0.32	4.88	0.42	3.34	0.44	-	-	5.10	0.50
Zr	-	-	-	-	-	-	3.55	0.50	-	-
Au	-	-	-	-	-	-	1.36	0.09	-	-

**Table 3:** Activation energies (*E*) calculated using Friedman method for bamboo, PE, and rice straw samples and their binary and ternary blends.

Sample/Conversion (*α*)	B100	P100	R100	B25R75	B50R50	B75R25	B25P75	B50P50	B75P25	R25P75	R50P50	R75P25	B25P25R50	B25P50R25	B50P25R25
0.05	194.987	183.044	228.873	152.808	153.709	192.846	304.699	176.863	163.922	181.399	162.357	135.330	97.843	130.788	160.246
0.10	190.611	228.938	226.722	185.807	167.462	194.705	-	196.253	163.899	194.971	184.789	151.595	111.641	144.818	166.450
0.15	195.770	240.203	238.501	185.506	177.264	207.030	-	211.399	169.522	162.417	188.167	150.032	110.275	148.710	165.273
0.20	198.071	265.393	238.087	184.925	179.677	211.673	-	203.550	163.930	87.487	185.344	151.588	112.526	155.624	165.160
0.25	197.589	295.553	241.859	186.456	177.157	214.083	-	190.868	171.151	155.276	197.743	144.735	112.752	192.197	163.197
0.30	204.797	308.560	238.077	180.807	176.598	208.793	-	217.428	179.280	186.013	220.487	139.706	110.387	188.287	167.973
0.35	204.196	289.467	239.280	179.431	171.767	205.572	205.672	231.758	186.954	200.642	217.319	124.550	107.529	-	182.200
0.40	205.169	288.734	233.809	177.277	173.009	188.683	221.148	-	170.287	204.790	-	105.121	98.960	130.630	181.257
0.45	208.857	274.678	228.388	176.481	176.867	188.616	218.702	186.281	169.873	212.528	163.703	-	77.127	190.997	195.652
0.50	217.425	297.955	238.814	178.270	171.883	196.819	230.337	186.102	161.227	219.516	193.086	-	-	198.702	176.606
0.55	198.222	286.885	240.624	185.333	181.638	228.942	224.205	201.188	162.524	226.704	200.625	-	71.199	210.242	161.440
0.60	205.359	299.706	240.144	192.696	187.368	231.422	223.266	214.819	170.871	231.541	215.377	-	-	218.050	148.852
0.65	194.454	258.173	256.379	208.482	176.891	211.773	202.789	224.586	158.327	262.315	221.978	77.591	-	222.571	186.470
0.70	183.047	259.639	278.643	234.870	178.591	211.283	211.914	225.580	221.636	217.092	225.051	210.457	187.178	228.566	211.535
0.75	187.131	239.370	-	239.066	202.869	212.733	219.788	252.174	224.392	222.964	231.223	271.097	243.642	227.958	225.631
0.80	174.710	207.020	-	293.192	237.954	214.111	257.612	255.836	246.912	218.561	253.108	282.266	244.172	234.468	221.916
0.85	215.276	201.992	-	-	-	238.206	248.228	244.732	262.859	217.594	239.296	291.225	249.035	233.707	234.403
0.90	-	255.061	-	-	-	236.702	241.667	275.869	272.805	210.556	250.882	329.127	259.797	238.299	284.640
0.95	-	245.881	-	-	-	-	258.947	-	-	-	303.479	-	300.229	264.794	329.642
Average (*E*_0_)	198.57	259.28	240.59	196.34	180.67	210.78	233.50	217.37	190.02	200.69	214.11	183.17	155.89	197.74	196.24

## Data Availability

Data will be made available on request.
